# Simultaneous Chromophobe renal cell carcinoma and squamous renal cell carcinoma

**DOI:** 10.1186/1746-1596-2-30

**Published:** 2007-08-21

**Authors:** Rafael Fúnez, Teresa Pereda, Isabel Rodrigo, Luis Robles, Carlos González

**Affiliations:** 1 Department of Pathology, Hospital Costa del Sol, Marbella, Spain

## Abstract

Chromophobe renal cell carcinoma (CHRC) is a neoplasm of the kidney with clinicopathologic peculiarities that seems to be of better prognosis than conventional renal cell carcinoma. Classical and eosinophilic types are the two histological variants recorded. Also, it has been described in association with carcinoma of collecting ducts, conventional renal cell carcinoma and sarcomatoid renal cell carcinoma. Squamous renal carcinoma is a very rare neoplasm with a malignant course. We describe a case of simultaneous chromophobe renal cell carcinoma with squamous cell carcinoma, finding which, to the best of our knowledge, has not previously been reported.

## Background

Chromophobe renal cell carcinoma (CHRC) is a neoplasm of the kidney described by Thoenes et al. in 1986 with clinicopathologic peculiarities, composed of typical cells with iron colloidal positive stain and seems to be of better prognostic than conventional renal cell carcinoma [1]. Classical and eosinophilic types are the two histological variants recorded. Also, it has been described in association with carcinoma of collecting ducts, conventional renal cell carcinoma and sarcomatoid renal cell carcinoma. We describe a case of concomitant chromophobe renal cell carcinoma and squamous cell carcinoma of the kidney.

## Case report

The patient, a 68-year-old hypertensive female presented with total hematuria and right flank pain. The ultrasonogragraphy demonstrated a solid mass with probably calcificated areas and cystic changes, and the CT scan a tumour in the upper pole of the kidney with heterogeneous contrast caption, calcifications and no enlargement of lymph nodes.

**Figure 1:**
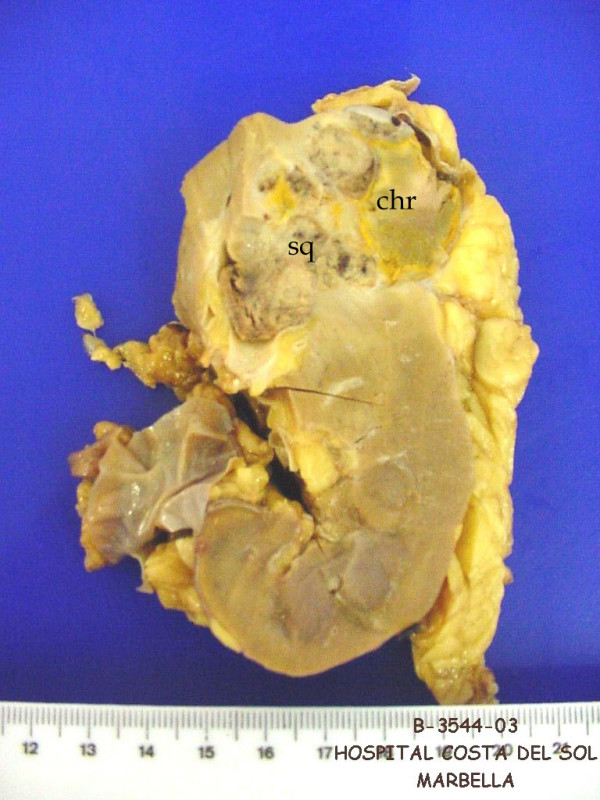
Macroscopical aspect of the neoplasm. Squamous cell carcinoma (sq) and Chromophobe renal cell carcinoma (chr).

Grossly the kidney showed a tumor centred in the upper pole with 4.6 cms in greatest dimension, without perinephric tissues or renal vein invasion. The neoplasm was in part brown and homogenous with other area grey coloured with necrotic/haemorrhagic appearance and calcifications. There was not presence of renal calculi in the renal pelvis. There was relationship of the neoplasm to the renal pelvis focally (figure 1). Also a normal macroscopically adrenal gland and two lymph nodes were received.

**Figure 2:**
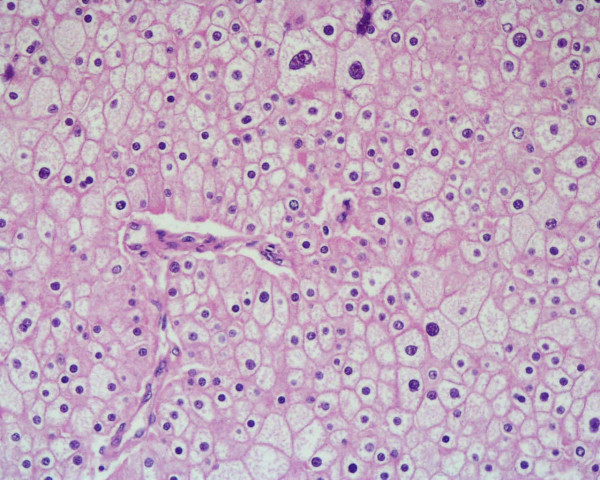
Areas with typical chromophobe renal cell carcinoma. HE × 200.

**Figure 3:**
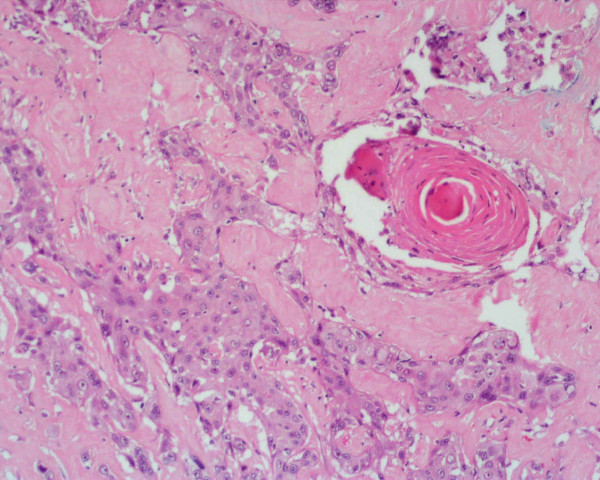
Zone with squamous cell carcinoma. HE × 200.

**Figure 4:**
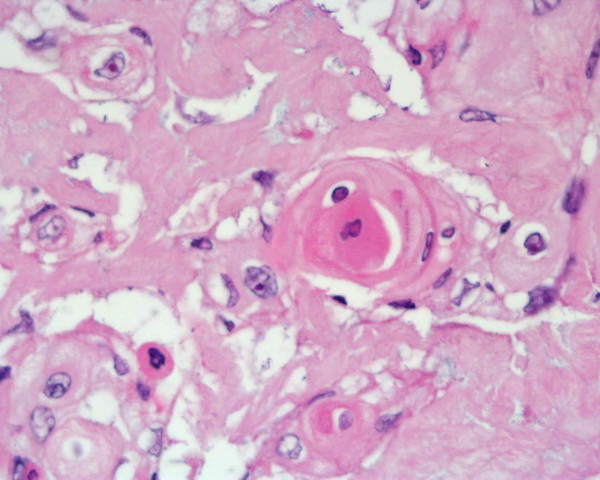
Zone with squamous cell carcinoma. HE × 400.

**Figure 5:**
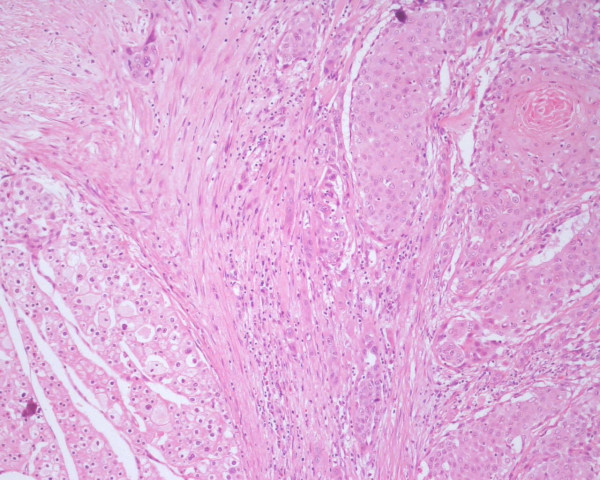
Border region of two tumors. There was not collission effect. HE × 100.

Microscopically the tumour was composed by a typical chromophobe renal cell carcinoma with positive Hale's colloidal iron stain and negative inmmunocytochemistry test for vimentin (Dakocytomation S.A.) in the areas with brown, homogeneous appearance (figure 2). Near by these areas there was another zone with necrosis, extensive calcification and solid epithelial nests with some keratin pearls and obvious squamous differentiation (figures 3, 4). These cells were negative for Hale's colloidal iron stain and positive for cytokeratin 5/6 (Dakocytomation S.A.). Sections from the area where both tumors were in contact did not show a collission effect (figure 5). There were focal relationship between both tumors and renal pelvis. We studied carefully the urinary tract to devoid an urothelial neoplasm and did not found signs of chronic tract infection or squamous metaplasia suggestive of irritation of pelvic or calyceal epithelium. The tumour did not invade perinephric tissues, adrenal gland or lymph nodes. The patient is alive without metastasis or recurrence after 32 months.

## Discussion

Transitional cell carcinoma constitutes the majority of the renal pelvis and calyces tumors. Squamous cell carcinoma accounts about 1% of renal neoplasms. A few cases of concomitant renal cell carcinoma and transitional cell carcinoma have been reported [2-4]. The simultaneous occurrence of renal cell carcinoma and squamous cell carcinoma is excepcional. The first case was reported by Elsa Valderrama et al in 1987. Subsequently, Charles et al described the association of renal squamous carcinoma and cystic renal cell carcinoma [3] and renal pelvis squamous cell carcinoma with renal cell carcinoma in a tuberculous kidney [4]. Likewise has been reported association of renal cell carcinoma with another renal cell neoplasms (conventional renal cell carcinoma, oncocytoma, collecting duct carcinoma) and cases with sarcomatoid transformation [5,6] or extensive calcification with ossification [7]. More recently also has been described one case with focal papillary growth pattern, basaloid features of the nuclear arrangement and stromal osseous metaplasia containing fatty bone marrow elements [8].

The histogenesis of the renal squamous carcinoma is controversial [2,9]. It is associated with renal pelvis calculi frequently, and continuos irritation of the transitional epithelium could produce metaplastic squamous changes and subsequently malignant transformation. CHRC is consistently positive for parvalbumin and calcium-binding protein expressed in the distal nephron, a feature further suggesting a histogenetic relationship between this tumor and the intercalated ducts [10]. Simultaneous chromophobe renal cell carcinoma and squamous renal carcinoma could present some questions. Perhaps, the most interesting of them is if the neoplasm is a CHRC with a concomitant squamous carcinoma derived from a transitional neoplasm, that seems more plausible, or if it is a squamous differentiation in a CHRC. It has to be said that the macroscopic appearance favor a unique motley neoplasm and the carefully study of the urinary tract did not show any urothelial neoplasm, urothelial displastic areas or any reason to justify irritation of the transitional epithelium. Curiously, Valderrama et al. did not found any of them in their case either [2]. Moreover, the squamous neoplasm were situated in a area with necrosis and extensive calcification, that could represent an strange form of differentiation later an event of tumoral necrosis. Anyway, this case it be either two concomitant different neoplasm or a differentiation in a CHRC, is a finding that has not previously been reported to the best of our knowledge.

## Authors' contributions

All authors read and approved the final manuscript.
